# P203: Outbreak of puerperal fever in an obstetric ward: a reminder of Ignaz semmelweis

**DOI:** 10.1186/2047-2994-2-S1-P203

**Published:** 2013-06-20

**Authors:** S Benenson, A Moses, I Gross, C Block, C Schwartz

**Affiliations:** 1 Clinical Microbiology and Infectious Diseases, Hadassah Hebrew-University Medical Center, Jerusalem, Israel; 2 Infection Control Team, Hadassah Hebrew-University Medical Center, Jerusalem, Israel

## Introduction

Group A streptococcus (GAS) puerperal fever was recognized long ago but unfortunately still poses a serious problem at childbirth. The source for the infection usually cannot be determined.

## Objectives

Description of the epidemiological investigation of an outbreak of "GAS" infection in women after childbirth, leading to recognition of the source of the outbreak. The Clinical Microbiology laboratory reported "GAS" isolated from vaginal specimens from two women readmitted shortly after delivery, one of whom also had a positive blood culture.

## Methods

An investigation was carried out to identify additional cases of "GAS" in the ward. All healthcare workers (HCWs) involved in taking care of the two women from admission till after delivery were identified, and requested to submit a throat swab. The specimens from the two women and from "HCWs" positive for "GAS" were compared regarding antibiotic susceptibility pattern, M protein gene typing (*emm* typing) and by Pulsed-field Gel Electrophoresis (PFGE).

## Results

No additional cases of "GAS" were found in the maternity ward. Both women were treated with intravenous antibiotics and one needed revision of the uterus.

A single "HCW", a midwife present at the deliveries of both affected women, had a positive throat swab. She reported having recently had a throat infection that was treated with antibiotics. *emm* typing of all four isolates showed them to be of the same type , emm 77, and their "PFGE" patterns were identical.

## Conclusion

Identification of identical strains of Streptococcus pyogenes from all specimens points at probable transmission from the midwife to both patients. We assume that the infection was transmitted by contaminated hands, although transmission by droplet or other indirect contact cannot be ruled out.

Ignaz Semmelweis recognized the role of hand hygiene in the prevention of puerperal fever 150 years ago and his observation is still relevant today.

## Disclosure of interest

None declared

